# Real-Time 0.89 THz Terahertz Imaging with High-Electron-Mobility Transistor Detector and Hydrogen Cyanide Laser for Non-Destructive Nut Detection

**DOI:** 10.3390/mi16020185

**Published:** 2025-02-04

**Authors:** Nu Zhang, Haiqing Liu, Huihui Yan, Hongbei Wang, Jiaxing Xie, Yinxian Jie, Damao Yao

**Affiliations:** 1 Institute of Plasma Physics, Hefei Institutes of Physical Science, Chinese Academy of Sciences, Hefei 230031, China; nu.zhang@ipp.ac.cn (N.Z.); huihui.yan@ipp.ac.cn (H.Y.); yx_jie@ipp.ac.cn (Y.J.); yaodm@ipp.ac.cn (D.Y.); 2 Science Island Branch, Graduate School of University of Science and Technology of China, Hefei 230026, China; 3 Institute of Energy, Hefei Comprehensive National Science Center, Hefei 230051, China; wanghb@ie.ah.cn (H.W.); jxxie@ipp.ac.cn (J.X.)

**Keywords:** real-time terahertz imaging, HCN laser, high-electron-mobility transistors, non-destructive inspections, nut detection, agricultural product detection

## Abstract

We present a method for real-time terahertz imaging that employs a hydrogen cyanide (HCN) laser as a terahertz source at 0.89 THz and an AlGaN/GaN high-electron-mobility transistor (HEMT) terahertz detector as a camera. We developed an HCN laser and constructed a transmission imaging system based on it. This combination utilizes a high-power HCN laser with a highly sensitive terahertz detector, enabling practical applications of real-time terahertz imaging. A resolution test plane was produced to determine that the system could achieve a lateral resolution of 2 mm, and real-time terahertz imaging was carried out on Siemens star, pistachios, and sunflower seeds. The results demonstrate that the hidden structures inside nuts can be observed by terahertz imaging. Through our analysis of terahertz images of both sunflower seeds and pine nuts, we successfully assessed their fullness and demonstrated the capability to distinguish between full and unfilled nuts. These findings validate the potential of this technique for future applications in nut detection. We discuss the limitations of the current setup, potential improvements, and possible applications, and we outline the introduction of aspherical lenses and terahertz transmission tomography.

## 1. Introduction

Over the years, terahertz imaging has been recognized as a potential tool for a variety of industrial solutions due to its ability to perform non-destructive and non-contact inspections [1,2,3,4]. Terahertz imaging technology has a good imaging effect in the presence of non-metallic materials, such as plastics, paper, clothing, wood, biological products, and other items [5,6,7,8]. Additionally, some terahertz or millimeter wave imaging technologies have been applied in security screening [9,10,11].

Despite the promising applications of terahertz imaging, in practice, this technology has remained in the laboratory context. Theoretically, terahertz imaging systems are simple, requiring only a terahertz light source, an optical imaging path, and a terahertz detector to form a complete terahertz imaging system. However, an entire terahertz imaging system must consider the power and wavelength of the terahertz source and the performance of the terahertz detector to achieve good results.

Two methods for real-time terahertz imaging are direct irradiation of the sample using a terahertz light source and obtaining a terahertz image through a focal plane detector, as well as single-pixel imaging [12]. Direct irradiation of the sample and acquisition of a real-time terahertz image through a focal plane detector offer a simpler and more efficient approach to real-time terahertz imaging compared to terahertz single-pixel imaging. The latter requires complex configuration and computational imaging to obtain a real-time terahertz image. Real-time imaging requires an intense THz source when active sample illumination is necessary. The spatial resolution of the image is directly related to the wavelength of the light used for illumination; therefore, the wavelength of the source is a crucial factor to consider.

Terahertz generation methods can be classified into two types: electronic and photonic methods [13,14,15,16,17]. Electronic methods consist of vacuum electronic devices and solid-state electronic devices. Examples of vacuum electronic devices include backward wave oscillators [15], traveling-wave tubes [18], and free-electron lasers [19,20]. Solid-state electronic devices are available in Gunn diodes [21], transistors [22], and frequency multipliers [23]. With the exception of free-electron lasers, electronic terahertz sources operate in the low-frequency terahertz band below 1 THz, and their output power decreases as the operating frequency increases, reaching approximately 1 mW at around 1 THz [15,24]. However, low-frequency terahertz waves have low resolution for imaging due to optical diffraction limitations. While free-electron lasers can operate in the frequency range above 1 THz, they are complex and expensive to fabricate. Photonic terahertz sources can be divided into two main types: terahertz lasers and terahertz optoelectronic devices. Terahertz gas lasers [25] and semiconductor lasers such as quantum cascade lasers are the main types of terahertz lasers [26,27]. The primary optoelectronic terahertz sources are photoconductive antennas [28], optical rectification [29], and difference frequency generation (DFG) [30]. Compared to other terahertz wave sources, gas laser-based terahertz wave-generating technology is more suitable for real-time terahertz imaging systems due to its high output power, structural simplicity, environmental flexibility, and low cost.

Terahertz detectors are available as field-effect transistors [31,32], microbolometers [33], pyroelectric detectors [34] and Golay cells detectors [35,36], Schottky mixers [37], photoconductive antenna [38], and others [39,40]. The current widespread usage of point-by-point terahertz imaging based on a single image element detector, resulting in a sluggish imaging speed, precludes high-speed, real-time terahertz video imaging. Terahertz focal plane array technology is currently transforming terahertz imaging, with several types of terahertz array cameras currently commercially available, including high-electron-mobility transistors (HEMTs), silicon complementary metal oxide semiconductor (CMOS) circuits, microbolometers, and pyroelectric devices. Terahertz focal plane cameras have enabled a significant improvement in the real-time nature of terahertz imaging.

Real-time terahertz imaging using Terahertz continuous wave (CW) discharge-pumped hydrogen cyanide (HCN) lasers and HEMT terahertz array detectors enables the acquisition of high-resolution terahertz images, which is not possible when using low-frequency sources. The HCN laser is a kind of THz laser working in 0.89 THz (337 μm), and the output power is up to 100 mW. Highly sensitive terahertz array detectors based on antenna-coupled AlGaN/GaN high-electron-mobility transistors are capable of high-speed imaging at room temperature. Recent terahertz imaging studies of nuts have been reported in the literature [41]; however, most have relied on photoconductive antenna imaging and terahertz time-domain spectroscopic imaging [42]. In contrast, the HCN laser we employ as a light source is capable of generating higher-power terahertz waves, enabling real-time terahertz imaging with significantly greater field-of-view scalability.

The proposed terahertz imaging system demonstrates distinct advantages over existing technologies by achieving high-resolution imaging with a large field of view at room temperature, while maintaining a balance between cost and system complexity. In contrast, quantum cascade laser-based systems typically require cryogenic cooling and incur substantial expenses [43], whereas terahertz time-domain imaging systems demand complex configurations, often necessitating mechanical delay lines or imposing strict femtosecond laser repetition rate requirements [4]. Moreover, compared to single-pixel imaging approaches, the proposed system achieves greater efficiency in utilizing both algorithmic and hardware resources.

This paper presents a real-time terahertz imaging system built using an HCN laser and a HEMT terahertz array detector. The devices, imaging method, and system setup are described. The lateral resolution and spatial resolution of the system were determined based on the imaging results of the Siemens star map. Additionally, real-time terahertz imaging experiments and subsequent image processing were carried out on various types of nuts, including sunflower seeds, pistachios, and pine nuts. The real-time terahertz images of the nuts were subjected to super-resolution and binarization techniques to enhance their resolution and assess their fullness. The results of these experiments and subsequent image processing highlight the potential applications of the system in various fields, such as agricultural product detection.

An earlier version of this paper was presented at the International Conference on Infrared, Millimeter and Terahertz Waves [44], and our initial conference paper did not analyze and process the image further. This manuscript addresses this issue and provides additional analysis on the nuts.

## 2. Setups and Methods

### 2.1. HCN Laser and Terahertz Detector

We built an HCN laser, which is a mature far-infrared gas laser with a parallel planar cavity structure. The resonant cavity is formed by a 3.4 m long, 54 mm diameter glass discharge tube and two plane reflectors against its ends. One end of the resonant cavity features a movable plane mirror with a gilded glass substrate, while the other end has a nickel mesh with 500 lines per inch (LPI) for coupled output, which reflects 93% of the incident radiation. The laser employs two brass electrodes at each end to connect the high voltage for electrode glow discharge. The HCN laser charges a gas mixture of N_2_:CH_4_:H_2_ in an optimal ratio of 1:1:5. During operation, high-voltage electrical excitation is applied to the cathode and anode at each end of the laser resonant cavity, causing collisional excitation of electrons in the gas molecules. The output power of the HCN laser for the EH_11_ mode is up to 100 mW at a wavelength of 337 μm.

The power supply utilized by the HCN laser for electrically excited glow discharge is a 10 kV high-voltage DC system. It has an input voltage of 380 V and an adjustable output current ranging from 0.2 to 1.5 A. The current stability control accuracy is ±0.5%, while the output voltage ranges from 1.9 kV to 6 kV, with a voltage stability control accuracy of ±0.1%. Additionally, the control response time for both output current and output voltage is within 1 µs. Due to its minimal output voltage ripple, the power supply ensures stable output voltage and current, as well as rapid response speed, thereby facilitating the long-term stable operation of the laser. When the HCN laser operates stably, the output voltage of the power supply is approximately 3.8 kV, and the output current is approximately 680 mA.

During the operation of the HCN laser, a significant amount of heat is generated, leading to changes in the length of the resonant cavity, which in turn affects the laser’s output power. To mitigate this issue, we utilized four Invar alloys (with a thermal expansion coefficient of 1.5 × 10⁻⁶ °C⁻¹) as the frame of the laser and connected a water-cooling circulation system to the heat-generating electrodes at both ends to suppress resonance cavity changes. Additionally, the length of the resonant cavity was adjusted using a movable mirror at one end to compensate for slight deformations, thereby ensuring the stability of the laser’s output power.

The terahertz detector utilized in this study is a plane array detector based on AlGaN/GaN high-electron-mobility transistors (HEMTs). The detector is a 32 × 32 pixel array with 200 μm spacing between each pixel and can detect terahertz waves in the 0.74–1.15 THz band. It can output images at a frame rate of 30 Hz at room temperature when exposed to 0.89 THz terahertz radiation. Compared to conventional terahertz detectors, the HEMT terahertz detector is capable of uncooled focal plane imaging and has an extremely low noise-equivalent power (NEP) of 50 pW/Hz^0.5^.

### 2.2. Experiment Setup

**Figure 1:**
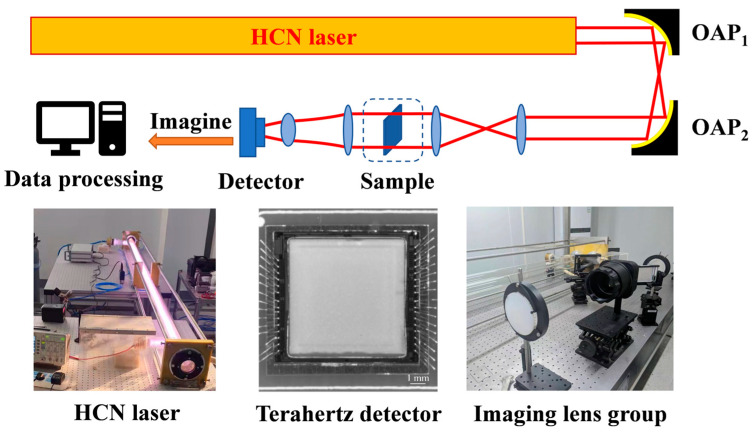
The structure of real-time THz imaging system. The collimated terahertz beam emitted from the HCN laser is first redirected by an off-axis parabolic mirror (OAP1 and OAP2) and then expanded through a dual-lens system to achieve an 80 mm illumination field. This expanded beam transmits through the sample and is subsequently focused onto a HEMT detector using a compound imaging lens assembly.

The scheme of the real-time imaging system built using the HCN laser and the HEMT detector is shown in Figure 1, while the second half shows the HCN laser, HEMT detector, and imaging lens group (with a focal length of 50 mm).

The optical system comprises three key components: a terahertz source, lighting and imaging optical path, and a data control receiving computer. The active light source in this system is an HCN gas laser built on the optical platform, which outputs a 0.89 THz terahertz wave. As previously mentioned, the HCN gas laser continuously produces terahertz waves at a consistent wavelength of 337 µm with a horizontal polarization direction and a beam waist diameter of approximately 21 mm at the exit port. Two off-axis parabolic mirrors initially guide terahertz light from the HCN laser, and the optical platform area adjacent to the laser is used to construct the terahertz imaging system. A set of terahertz lenses made of high-density polyethylene (HDPE) material expands and collimates the terahertz light, increasing the initial 21 mm spot to approximately 80 mm in diameter. The light is then collimated to parallel light for transmission imaging. The parallel terahertz light used to identify the item is directed to the measurement region and passes through the sample. The transmitted light then passes through a set of imaging lenses, which consist of high-density polyethylene and TPX lenses. The focal length of the imaging lens is approximately 50 mm, and the object to be measured forms a reduced inverted actual image on the detector chip with an imaging magnification of 0.1.

The primary challenge in constructing the overall optical path lies in accurately determining the positions of the devices within it. When the lenses or parabolic mirrors are misaligned, the illumination spot detected by the HEMT detector is weakened, resulting in significant diffraction. To facilitate the adjustment process of the optical path, a He-Ne laser, with the output of the light collineated with the HCN laser, is used as a reference.

Inside the HEMT terahertz detector, a terahertz antenna is utilized to induce an enhanced terahertz electric field parallel and perpendicular to the trench. The nonlinear effect of the field-effect tube on the trench conductance regulation results in the self-mixing of the two induced terahertz electric fields at the trench, which produces a photocurrent or photovoltage response proportional to the incident terahertz wave at both ends of the source-drain. The detector chip then responds to the terahertz wave by self-mixing and transmits the signal to the back-end circuit through the readout circuit. The analog signal output from the array sensor is initially converted into a digital signal by the back-end circuit. Subsequently, the digital signal is fed to a computer using an acquisition circuit comprising a field-programmable gate array (FPGA) [45].

## 3. Results

### 3.1. System Resolution

**Figure 2:**
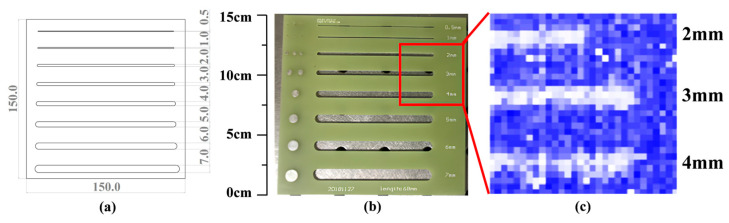
Terahertz imaging results of a resolution test plate. (**a**) is the design diagram of the resolution test plate, and the size of the test plate is 150 mm × 150 mm. From top to bottom, there are slots ranging from 0.5 mm to 7 mm, which are used to test the line resolution through terahertz light. (**b**) is a visible light image of the test plate, and (**c**) is the terahertz image corresponding to the red box position in (**b**).

To measure the system’s resolution, a resolution test plate was created and placed in the imaging inspection area. Figure 2 shows a photograph of the plate, whose size is 150 mm × 150 mm. The plate features eight slots with a total length of 100 mm, each located 10 mm apart. The width of the slots increases from 0.5 mm to 1 mm, 2 mm, and 3 mm, with the largest slot measuring 7 mm. The resolution test plate is positioned in the detecting location of the transmissive terahertz imaging system, and a real-time terahertz image is obtained.

We adopt the geometry of the ISO-12233 standard [46] test chart, selecting parallel line pairs and Siemens star charts to evaluate the resolution of the imaging system. The chosen geometry is based on two key principles: (a) covering a spatial frequency range that exceeds the Nyquist frequency of the system and (b) incorporating known dimensional features to enable absolute scale calibration. The parallel line pairs feature lines of a specified width, allowing for a precise assessment of resolution. In this study, we leverage the versatility of parallel line pairs to specifically evaluate the lateral resolution of the imaging system. Notably, Siemon stars have been extensively employed as resolution testing tools in X-ray imaging and optical coherence tomography, demonstrating their efficacy in characterizing imaging system performance.

The bright and dark areas in the terahertz image correspond to the slots and plate of the resolution test plate, respectively. This is due to the reduced transmission of the resolution test plate material in the terahertz band. The terahertz image obtained by the transmission system clearly displays the smallest slot, which measures 2 mm. The shape of the slot is easily identifiable through visual inspection of the terahertz image. Figure 2b shows the visible light image, and Figure 2c shows the terahertz imaging results. The red box in Figure 2b correspond to the terahertz imaging results for 2 mm, 3 mm, and 4 mm slots in Figure 2c.

**Figure 3:**
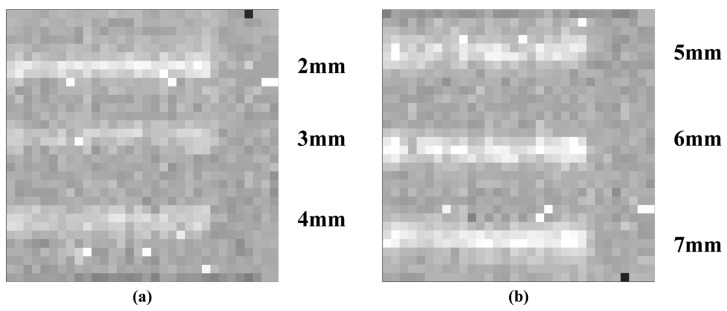
Terahertz images of resolution test plates with 2–7 mm slots. (**a**) show the terahertz imaging results for 2–4 mm slots, and (**b**) show the terahertz imaging results for 5–7 mm slots.

As the imaging area of the transmissive real-time imaging system is limited to 60 mm × 60 mm, the resolution test plate must be moved to perform transmissive imaging for each of the different slot widths. Figure 3 displays the resulting images. By moving the resolution test plate, it is possible to observe the terahertz images corresponding to each inscribed slot, ranging from 2 mm to 7 mm. Figure 3a shows the terahertz imaging results for 2–4 mm slots, and Figure 3b shows the terahertz imaging results for 5–7 mm slots. This demonstrates the transmission terahertz imaging system’s ability to achieve a lateral resolution of 2 mm. The speed of movement of the resolution test plate during imaging is approximately 10 cm/s.

**Figure 4:**
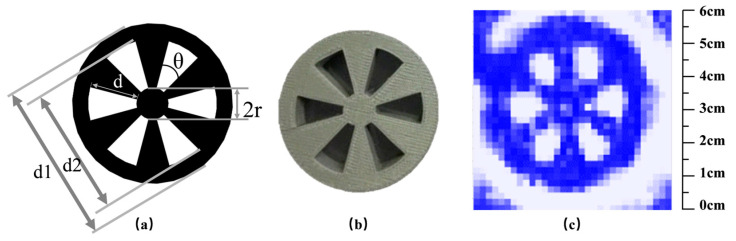
Imaging results from the Siemens star map. (**a**) is the design diagram of Siemens Star, (**b**) is the visible light image of Siemens Star, and (**c**) is the corresponding terahertz image.

To quantitatively assess the spatial resolution of the imaging system, we conducted tests using the Siemens star [47], a tool commonly employed for evaluating the spatial resolution of optical systems across a broad frequency range [48]. Figure 4a–c illustrates the design of the Siemens star, visible imaging, and real-time terahertz imaging, respectively. The Siemens Star Chart is a device used to test the resolution of optical instruments, printers, and displays. It consists of a pattern of bright spokes on a black background, which radiate from a common center and grow wider as they move away. The theoretical Siemens chart features six spokes that only meet at the emission center, with the gap between the spokes narrowing from the outside to the inside, closer to the center.

The Siemens star is a circle with an outer diameter (d1) of 50 mm, an inner circle diameter (d2) of 40 mm, and a center diameter (d) of the linked spokes of 10 mm. This is illustrated in Figure 4a. To enhance the contrast of the terahertz image, a three-dimensional Siemens star map plate with a thickness of 10 mm was printed on a 3D printer using polylactic acid (PLA), as shown in Figure 4b. When terahertz light passes through the Siemens star map plate, a high-contrast image can be obtained. The Siemens star plate is positioned in the detection location of the terahertz imaging system depicted in Figure 4, and real-time terahertz images are obtained by adjusting the front-to-back distance, as shown in Figure 4c.

The periodic structure shown in Figure 4a belongs to a well-known class of resonant geometries that have been extensively studied across different frequency ranges. In vacuum electronics, similar structures are employed in slow-wave circuits for traveling-wave tubes (TWTs) and klystrons, where they facilitate efficient electron beam-wave interaction at GHz frequencies [49]. At optical frequencies, periodic metallic structures are used to excite surface plasmon polaritons (SPPs), enabling subwavelength light confinement and enhanced field localization [50]. These structures, often referred to as metasurfaces, have found applications in sensing, imaging, and energy harvesting.

**Figure 5:**
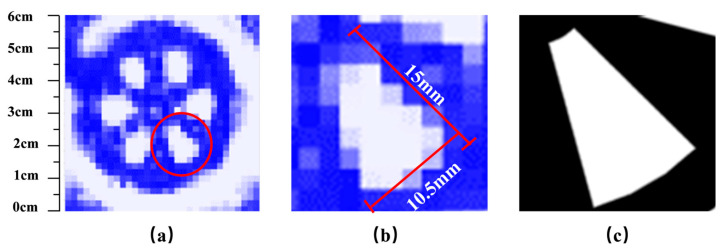
Real-time terahertz imaging of a slit in a Siemens star. (**a**) display the real-time terahertz image of the Siemens star plate obtained through the transmission terahertz imaging system. The red circles in (**a**) highlight the spokes, and the sector ring image of the spokes is shown in (**c**). (**b**) displays the obtained terahertz image, which successfully restores the structure of the sector ring.

Figure 5a displays the real-time terahertz image of the Siemens star plate obtained through the transmission terahertz imaging system. The outline of the Siemens star plate is clearly visible, and the shape of the spokes and outer circular outline is well defined. The red circles in Figure 5a highlight the spokes, and the sector ring image of the spokes is shown in Figure 5c. Figure 5b displays the obtained terahertz image, which successfully restores the structure of the sector ring. By comparing with the perimeter of the outermost edge of the Siemens star, the length of Figure 5b edge can be measured as 15 mm, and the length of the bottom curve is about 10.5 mm. However, due to the resolution limit of the detector array, which consists of 32 × 32 image pixels, the original straight and arc shapes appear jagged in the 60 mm × 60 mm imaging area. Nonetheless, the overall structure is preserved. The terahertz imaging results in Figure 5 depict unprocessed raw data, and no spatial filtering or other image processing techniques were employed to avoid compromising the spatial resolution. During real-time imaging, rotating the Siemens star plate is effective in demonstrating the imaging effect since the human eye is more sensitive to moving objects. In addition, the shaded area extending from the Siemens star plate in the upper left corner of Figure 5a is the image of the clamped object, not the Siemens star plate itself. Based on the equation r_res_ = 2π∙r_min_/N, where r_min_ is the smallest identifiable radius and N is the number of spokes, the spatial resolution of the system is estimated to be r_res_ = 5.23 mm. Spatial resolution is the smallest object size that can be distinguished in an image. Given that the maximum outer diameter of the Siemens star (d1) is known, we first fit the smallest identifiable circle at the center of the Siemens star within the terahertz image. In this image, when the intensity difference between adjacent radial lines at the center of the Siemens star is less than 3 dB, the corresponding region is identified as the smallest identifiable circle. The diameter (2r) of this circle is then compared to the pixel positions corresponding to the maximum outer diameter (d1) of the Siemens star in the image. Based on the proportional relationship between the actual dimensions and the pixel positions, the actual value of 2r is calculated. This allows the smallest identifiable radius (r) to be determined. Axial resolution (depth resolution) is generally lower than lateral resolution (transverse resolution) due to the inherent limitations of coherence length and depth-of-field in imaging systems.

### 3.2. Real-Time Imaging of Different Agricultural Products

A nut is a type of indehiscent fruit that has a hard skin enclosing one or more seeds. The oil content of the nut is indicative of its maturity and ripeness. While traditional inspection methods are unable to non-destructively detect nuts, terahertz imaging enables visualization of the seeds within the nut without causing damage. For our experiments, we selected pistachios and sunflower seeds as they are widely used in both daily life and industry.

We utilized nuts measuring between 15 and 20 mm in length and 10 mm or less in width as specimens for our imaging trials. These specimens were bifurcated into two groups for comparative analysis—one containing the complete nut and the other with the shell detached from the kernel. This comparison approach was employed to evaluate the imaging prowess of the terahertz imaging system.

**Figure 6:**
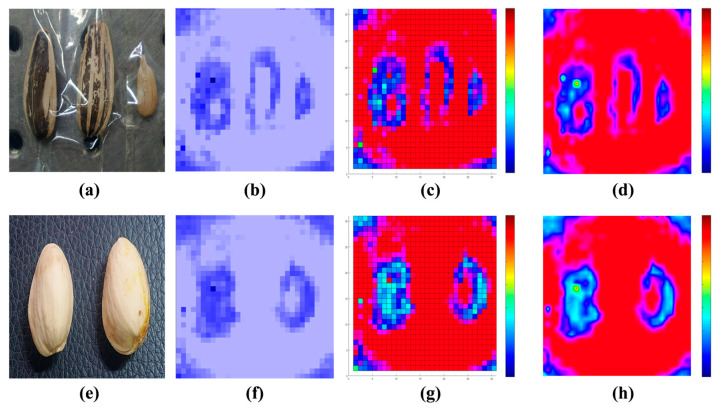
(**a,e**) depict the visible light images of a sunflower seed and a pistachio, respectively. (**b**) shows the real-time terahertz imaging result of the sunflower seed. While hollow and plump sunflower seeds cannot be distinguished in the visible light image, they can be clearly differentiated using terahertz imaging. To enhance the contrast and resolution of the image for further investigation, pseudo-color processing was applied to the raw terahertz image. (**c**) presents the pseudo-color-processed image of the sunflower seed. (**d**) displays the terahertz image of the sunflower seed after super-resolution processing. (**f**) illustrates the real-time terahertz imaging result of the pistachio, revealing that the right pistachio exhibits higher transmittance, indicating it is actually a defective nut. (**g**) shows the pseudo-color-processed image of the pistachio. (**h**) provides the terahertz image of the pistachio after super-resolution processing.

Figure 6 shows terahertz imaging of pistachios and sunflower seeds. Figure 6a,e show visible light images of sunflower seeds and pistachios, respectively. The sample in Figure 6a shows a sunflower seed, an empty shell, and a sunflower kernel, and the sample in Figure 6e shows a pistachio and an empty shell. However, visual inspection does not distinguish between a good nut and an empty nut shell. Terahertz images of both samples are shown in Figure 6b,f. It can be seen that the empty shell without a nut kernel only shows the outline of its shell in the terahertz image, while the terahertz image of the good nut produces a shadow in the center region. By comparing the results of the two terahertz transmission images, the product quality can be determined in real time.

To further analyze the features of the terahertz images of the samples, the acquired images underwent additional image processing. To enhance the contrast and resolution of the image for further investigation, pseudo-color processing was applied to the raw terahertz image. The grayscale map was extracted from the acquired real-time terahertz video. The grayscale distribution was then divided into three groups (0~255): 0~85, 86~170, and 171~255. The grayscale was classified according to the three groups based on the grayscale level, and the grayscale values of the three channels were saved in three different matrices. Each channel was assigned a different mapping function for the grayscale values. Specifically, low grayscale values were mapped as blue, middle grayscale values as green, and high grayscale values as red, resulting in the pseudo-color map corresponding to the grayscale shown in Figure 6c,g.

The terahertz detector used in this study has pixels of 32 × 32, resulting in a maximum of 1024 pixels. This system allows for the differentiation of the cavity in the nut. However, the resolution of this system is relatively low compared to visible images, and super-resolution processing is required for further and more accurate analysis of the sample shape in subsequent studies. Although the existence of the hollow inner region can be visually observed, the small size of the sample content is inadequate for accurate judgment of the specific shape of the hollow area, leading to potential misjudgment. Therefore, the image was processed with super-resolution using the interpolation method, resulting in a 1024 × 1024 resolution image shown in Figure 6d,h. The processed images display a smooth edge curve and accurately depict the shape of the shell and kernel outline, resembling the original images.

**Figure 7:**
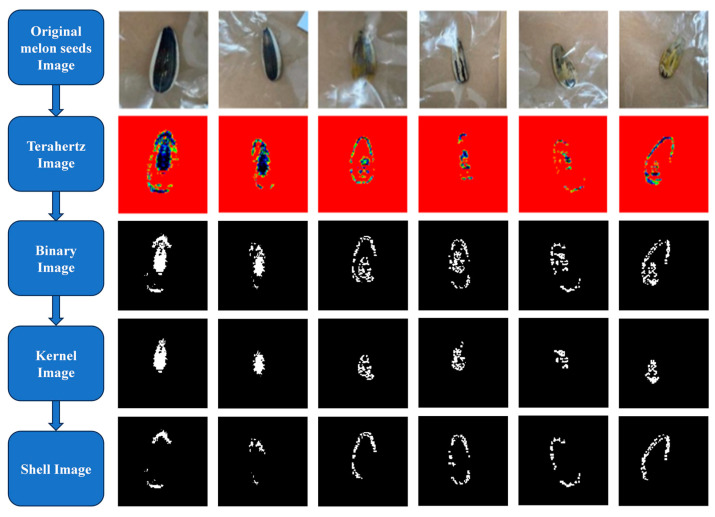
Terahertz image processing flow of sunflower seeds. From top to bottom is the processing process of terahertz images of six sunflower seeds. At the top is a visible image, followed by a terahertz image from the terahertz imaging system. Then, the terahertz images were binarized and segmented into sunflower seed shell and kernel images.

**Figure 8:**
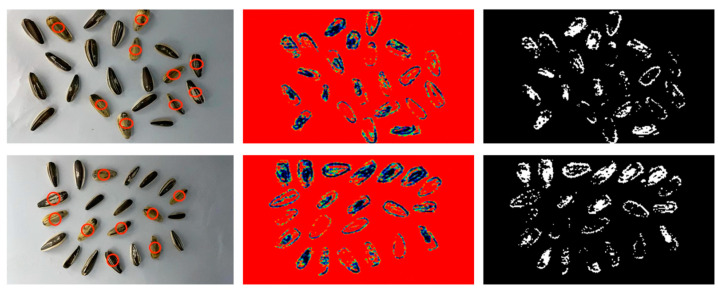
Terahertz images of batch sunflower seeds and their binarized images. On the left are visible images of two piles of sunflower seeds, with red circles indicating bad fruit; the middle image is the result after false color processing, and the right image is the result after binarization processing.

Terahertz transmission imaging was conducted on six sunflower seed samples with varying qualities, as shown in Figure 7. Image processing methods were employed to assess the integrity of the sunflower seed samples. Figure 8 illustrates the real-time flow of terahertz transmission image processing for the sunflower seed samples. The top row of Figure 8 presents the visible light imaging of the six samples. The second row displays the real-time terahertz transmission imaging results of sunflower seeds. It can be observed from the images that the terahertz images of the first two samples not only depict the outer contour but also reveal the shape of the center fullness of the sunflower seed kernel. However, the terahertz images of the remaining four samples only exhibit the sparser shape of the sunflower seed kernel within the contour. This suggests that the fullness of the sunflower seeds can be characterized by the ratio of the sunflower seed kernel region to the overall bright spot region in the image. The third row presents the binarized image, which was generated using MATLAB software (v.24.1.0) with a threshold scale set at 0.29. This step enables clearer visualization of the internal structure of the sunflower seeds.

The binarized image underwent processing to distinguish between sunflower seed shells and kernels. The fourth and fifth rows of the image in Figure 8 depict the processed kernels and sunflower seed shell images, respectively. To determine the fullness of sunflower seeds, we calculated the proportion of the kernel within the sunflower seeds in the image. The corresponding calculated results show the indicated fullness percentages of 66%, 70%, 30%, 39%,42%, and 46%. It is evident that high-quality sunflower seed samples exhibit a significant degree of fullness, often surpassing 60%. Conversely, low-quality sunflower seed samples typically display a lower degree of fullness, usually below 50%.

### 3.3. Terahertz Imaging Results of Bulk Sunflower Seeds and Pine Nuts

The previous section demonstrates the capability of the real-time transmission terahertz imaging system to transmit images of individual pistachio and sunflower seed samples, acquire real-time imaging results, and differentiate between different quality levels of the samples. Building upon these findings, it is possible to expand the field of view in order to detect a greater variety of products, thereby extending the system’s range of applicability. For testing purposes, both sunflower seeds and pine nuts were selected, with approximately 20 samples used for each test. Due to the terahertz imaging system’s output frame rate of thirty frames, we were able to obtain real-time imaging results by swiftly scanning the samples.

Figure 8 presents the experimental results for sunflower seed samples. In the first column, visible light images of sunflower seed samples are displayed, with samples exhibiting hollow sunflower shells or unfilled inner kernels marked by red circles. The second and third columns showcase their corresponding terahertz and binarized images, respectively. The terahertz imaging results of the two sample groups reveal distinct differences between the unsaturated sunflower seed samples and the full samples after the binarization process. Upon segmenting the samples in the binarized image and calculating their fullness separately, the full samples exhibited a fullness of over 60%, whereas the unsaturated samples displayed a fullness of less than 40%. In the case of hollow sunflower seed samples, the binarized image primarily contained outer contours and a few spots, resulting in a fullness of over 95% or less than 5%. This stark difference allows for clear differentiation between hollow sunflower seed samples and good sunflower seed samples.

**Figure 9:**
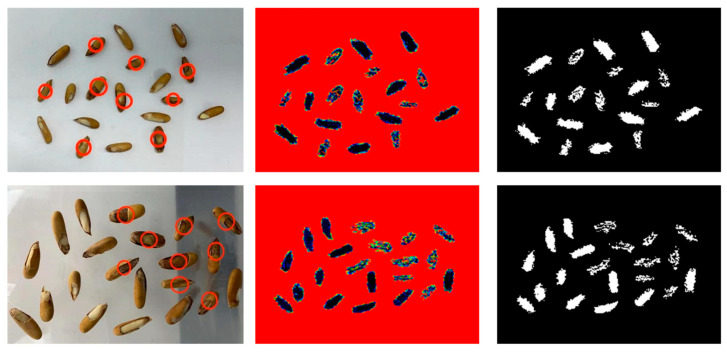
Terahertz images of batch pine nut seeds and their binarized images. On the left is a visible image of two piles of pine nuts, with the red circle marking the bad fruit. The middle image is the result after false color processing, and the right image is the result after binarization processing.

Figure 9 displays the experimental results for pine nut samples. The first column presents the visible light image of pine nut samples, with unsaturated pine nut samples identified by red circles. The second and third columns feature their respective terahertz and binarized images. It is evident that the terahertz images of pine nut samples in the second column do not effectively differentiate the fullness of pine nuts based on their morphology. However, upon binarizing the terahertz images, a noticeable distinction between full and unsaturated samples emerges. The binarized image obtained for full pine nut samples appears continuous, whereas the binarized image obtained for unsaturated pine nut samples contains holes. Therefore, this approach enables rapid differentiation between good- and poor-quality pine nut samples, facilitating the effective identification of defective pine nuts.

## 4. Discussion

### 4.1. Limitations and Potential Improvements

Terahertz imaging that employs a combination of an HCN laser and a HEMT detector facilitates the efficient utilization of a powerful terahertz source, enabling real-time imaging. However, because the imaging system operates as a quasi-optical setup and the terahertz waves emitted by the HCN laser are Gaussian beams, the intensity of the light diminishes with the square of the beam radius [51]. Consequently, the signal-to-noise ratio of the resulting image will be significantly higher in the central region compared to the edges.

The spatial resolution and imaging area of the system are predominantly constrained by the detector’s specifications. In this study, the detector resolution is limited to 32 × 32 pixels. As benchmarked against comparable imaging systems (Table 1), significant improvements in resolution could be achieved by integrating a higher-resolution detector. The HCN laser employed in the system operates at a wavelength of 337 μm. Notably, the current imaging resolution remains far below the theoretical diffraction limit, suggesting substantial headroom for optimization. Concurrently, the laser’s output power is sufficient to support a doubling of the field of view (FoV). Thus, future enhancements—such as upgrading the detector resolution while refining the optical design—could enable simultaneous improvements in both resolution and FoV. Specifically, the FoV could be expanded to 100 mm × 100 mm or larger while achieving sub-1 mm resolution.

In addition to the influence of the light source, the quality of the images produced by the imaging system is significantly affected by the imaging lens. Objective lenses are critical optical components in a high-quality imaging system; however, standard spherical lenses, which typically comprise objective lens groups, are not very effective at correcting aberrations generated in the optical path. The development of aspherical lenses for imaging detection presents an opportunity to further enhance resolution and optimize image quality at the edges [54,55]. An effective focusing system can expand the imaging range and simplify system complexity [49]. By optimizing the focusing system, the final imaging range and depth of field can be further improved.

To obtain high-resolution THz images, super-resolution imaging has become a research hotspot. Interpolation-based methods leverage the information from neighboring pixel points to estimate the value of an unknown pixel in a high-resolution image. The super-resolution of terahertz images through deep learning can enhance both the processing speed and the quality of the resulting images compared to conventional methods. This approach represents one of the potential strategies for further improving image quality in the future.

### 4.2. Potential Applications

The ability to image nut samples presents a promising application direction in the agriculture and food industries [41,42]. This imaging method enables early quality screening of produce post-harvest, particularly for high-quality or high-oil-content nuts. Additionally, it facilitates real-time inspection of products during production and transportation, as well as immediate spoilage detection of encapsulated products through their outer packaging.

Three-dimensional reconstruction of terahertz images represents a significant application for the enhanced utilization of terahertz imaging data. The implementation of array detectors facilitates higher data collection speeds, leading to the development of continuous wave terahertz holography and terahertz tomography. Terahertz holography offers the advantage of high spatial resolution, enabling the acquisition of amplitude and phase distributions without the need for scanning. The amplitude and phase information of an object’s wavefront is reconstructed by capturing the diffracted field information of the object or the interference pattern formed between the object and a reference beam, followed by digital numerical computation. In addition, terahertz transmission tomography is similar to the computer-aided tomography of X-rays, which utilizes the penetrating nature of terahertz waves for some media and can be applied to three-dimensional imaging of the internal structure of an object.

### 4.3. Outlook: Possible Modifications

Based on the terahertz images obtained, the imaging optical path must be optimized before acquiring further high-quality images. First, the illumination beam from the HCN laser is collimated and shaped simultaneously to homogenize the light intensity of the Gaussian-distributed beam. This adjustment can enhance image quality at the edges without reducing the signal-to-noise ratio in the central region. Second, introducing an aspherical lens into the imaging lens assembly helps eliminate spherical aberration, further contributing to the improvement of image quality.

Furthermore, a terahertz imaging system that combines a high-power terahertz light source with an HEMT detector enables the study of terahertz transmission tomography. Transmission images of the sample can be obtained by rotating it at various angles to reconstruct a three-dimensional image. Since the surface array detector captures data in the vertical direction during a single imaging session, the tedious process of scanning multiple heights is eliminated, significantly reducing the overall imaging time. Consequently, this advancement upgrades the information generated by the system from 2D to 3D images. HEMT detectors do not match the high lateral resolution of thermal microbolometers in terms of pixel count and spacing [34]. Thermal microbolometers, which feature a higher pixel density, are well suited for terahertz holography, while HEMT detectors are more appropriate for terahertz tomography.

## 5. Conclusions

We present a real-time terahertz imaging system comprising an HCN laser and a HEMT terahertz detector with 32 × 32 pixels. This combination offers a simpler structure, better environmental adaptability, and greater stability compared to other real-time imaging systems. The system also provides higher resolution than the low-frequency terahertz source approach, as well as a simpler structure and better real-time performance than the single-pixel imaging approach. To validate the system, real-time terahertz imaging experiments were conducted at a frame rate of 30 Hz. The system’s linear and spatial resolutions were determined using a resolution test plate and a Siemens star, with the line resolution of the system reaching up to 2 mm. Terahertz transmission imaging was employed to identify and analyze nuts and sunflower seeds, resulting in terahertz images representing hollow and full states. Pseudo-color and super-resolution processing techniques were applied to enhance the obtained images for subsequent studies. The high illumination intensity of the imaging system allows for an extended field-of-view size in real-time imaging, making it suitable for the real-time detection of nuts. Binary images were generated from terahertz images of individual and batch samples of sunflower seeds and pine nuts to differentiate the shell from the kernel. The hull-to-core ratios were then utilized to assess the fullness of the nuts. This approach demonstrates the system’s capability to be employed in diverse applications for detecting the abundance and conducting rapid screening of nuts. We discussed possible improvements and (practical) applications of the imaging system, including introducing an aspherical lens and terahertz transmission tomography.

## Figures and Tables

**Table 1:** Summarizing improvements over earlier THz systems.

Parameter	This Work	Zolliker et al. (2021) [28]	Perraud et al. (2020) [52]	Yang et al. (2023) [53]
Resolution	2 mm	1 mm	80	30 μm
Pixel of Detector	32 × 32	32 × 32	240 × 320	512 × 1024
Max Sample Area (mm²)	60 × 60	30 × 30	34 × 20	5 × 5
Imaging Time	Real-time	Real-time	Not real-time	Real-time

## Data Availability

Dataset available on request from the authors.
